# Herpes Zoster Ophthalmicus With Orbital Inflammation and Viral Meningitis in an Immunocompetent Patient

**DOI:** 10.1155/crop/4822819

**Published:** 2025-10-10

**Authors:** Khizar Rana, Jessica Y. Tong, Sumu Simon, Dinesh Selva

**Affiliations:** ^1^Department of Ophthalmology & Visual Sciences, University of Adelaide, Adelaide, Australia; ^2^South Australian Institute of Ophthalmology, Royal Adelaide Hospital, Adelaide, Australia

## Abstract

Common ocular manifestations of Herpes zoster ophthalmicus (HZO) include conjunctivitis, keratitis, anterior uveitis and ocular hypertension. Immunosuppressed patients are at an increased risk of sequelae including orbital inflammation and meningitis. We present a case of a 47-year-old immunocompetent patient who presented with acute orbital inflammation and viral meningitis, on a background of recent HZO without ocular involvement. Magnetic resonance imaging (MRI) demonstrated enhancement and enlargement of the left extraocular muscles, lacrimal gland and optic nerve sheath and enhancement of the left trigeminal nerve in the prepontine segment extending into the cavernous sinus. A lumbar puncture revealed elevated mononuclear cells consistent with viral meningitis. Cerebrospinal fluid polymerase chain reaction was negative for viruses. The patient was initially treated with intravenous acyclovir, which was followed by oral acyclovir. Oral prednisolone was commenced to treat the orbital inflammation. On 10-week follow-up, inflammatory signs had improved; however, the patient had persisting diplopia, which was managed with prismatic correction.

## 1. Introduction

Herpes zoster ophthalmicus (HZO) results from reactivation of latent varicella zoster virus (VZV) in the ophthalmic division of the trigeminal nerve. Common manifestations of HZO include keratoconjunctivitis, blepharitis and iritis. Immunosuppressed patients are at an increased risk of presenting with HZO-related orbital and neurological complications [1]. We present a case of an immunocompetent patient presenting with an acute orbital inflammatory syndrome with associated trigeminal neuritis and meningitis due to HZO.

## 2. Case Presentation

**Figure 1:**
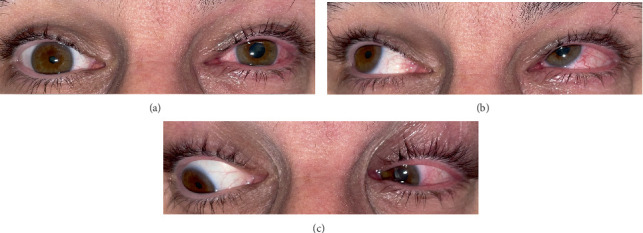
(a) There is mild periorbital oedema and conjunctival injection on the left and left-over-right hypertropia with anisocoria. Ocular motility was suggestive of (b) left inferior oblique overaction and (c) left superior oblique underaction.

A 47-year-old male presented with diplopia, left orbital pain and headache. Two weeks prior, he had been admitted with HZO without ocular involvement. He was treated with oral antivirals, and his skin lesions had resolved. He had no other medical conditions. On examination of the left eye, positive findings included diffuse conjunctival injection and mild periorbital oedema (Figure 1). Extraocular movements revealed a left-over-right hypertropia in primary gaze and left superior oblique underaction, mimicking a left trochlear palsy (Figure 1). There was reduced sensation in the left V_1_ and V_2_ dermatomes. Other examination findings, including visual acuity, colour vision and fundoscopy, were normal. Apart from a headache, he was otherwise systemically well.

**Figure 2:**
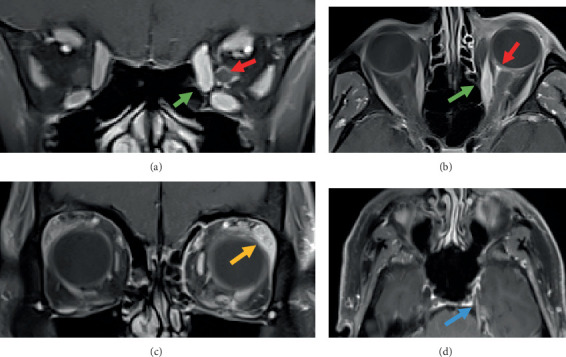
Fat-suppressed contrast-enhanced MRI demonstrating (a, b) enlargement and enhancement of the left medial rectus, inferior rectus and superior oblique muscles (green arrows), (a, b) enhancement of the left optic nerve sheath (red arrows), (c) lacrimal gland enhancement and enlargement (orange arrow) and (d) enhancement of the prepontine left trigeminal nerve (blue arrow).

Magnetic resonance imaging (MRI) revealed more prominent enhancement and enlargement of the left medial rectus, inferior rectus and superior oblique muscles compared to noninvolved muscles, enhancement of the left optic nerve sheath suggestive of optic perineuritis, more prominent lacrimal gland enhancement and enlargement and enhancement of the left trigeminal nerve in the prepontine segment extending into the cavernous sinus (Figure 2). In view of these radiological changes, a lumbar puncture was performed and revealed elevated mononuclear cells (105 × 10^6^, reference range 0–5 cells) consistent with viral meningitis. Oral prednisone 1 mg/kg was commenced once the cerebrospinal fluid (CSF) polymerase chain reaction (PCR) was reported as negative for viruses. The patient was treated with a weaning course of oral prednisone and intravenous acyclovir for 2 weeks, which was followed by oral acyclovir for another week. On 10-week follow-up, inflammatory ocular signs had improved; however, the patient had persistent diplopia, which was managed with prismatic correction.

## 3. Discussion

Common ocular manifestations of HZO include conjunctivitis, keratitis, anterior uveitis and ocular hypertension. Immunosuppressed patients are at an increased risk of developing inflammatory sequelae in the orbit and intracranially. Our case demonstrates the potential for HZO to present with widespread orbital inflammation extending into the prepontine trigeminal nerve and meninges in an immunocompetent patient.

Differential diagnoses include cavernous sinus thrombosis, orbital cellulitis and idiopathic orbital inflammation. Cavernous sinus thrombosis usually shows venous sinus filling defects on head venogram, whilst orbital cellulitis typically presents with ophthalmoplegia, proptosis, chemosis and sinus disease on imaging. Idiopathic orbital inflammation can involve any part of the orbit, but, given the temporal link with zoster rash and trigeminal enhancement, HZO was favoured in our case.

HZO presenting with a simultaneous acute orbital inflammatory syndrome and neurologic manifestations is rare. Orbital manifestations of HZO include myositis, optic perineuritis or dacryoadenitis. In HZO myositis, the presentation is ipsilateral to the rash and can involve any of the extraocular muscles, including the lateral rectus. Radiologically, the anterior tendon is, however, usually spared, similar to TED [2]. Some patients with orbital myositis may not present with diplopia and have normal extraocular movements, despite radiographic evidence of myositis [3]. Our patient had myositis with resultant left hypertropia mimicking a fourth nerve palsy.

Other orbital manifestations include dacryoadenitis and optic perineuritis. Optic perineuritis was diagnosed radiologically in our case, without clinical evidence of optic nerve dysfunction. This potentially represented an early phase of optic perineuritis, signifying the need for prompt treatment to prevent visual compromise. Clinically, the diagnosis of dacryoadenitis may be obscured by the surrounding orbital inflammation and optic neuritis. Radiologically, enhancement and enlargement of the lacrimal gland can be seen as in our case. It is potentially reversible with antiviral treatment, although routine follow-up imaging is generally not required [4].

Our case was complicated by VZV meningitis and trigeminal neuritis. MRI showed enhancement of the prepontine trigeminal nerve, and this correlated clinically with the reduced sensation in the trigeminal nerve branches. A lumbar puncture was performed due to the patient's headache and neuroimaging showing extensive inflammation extending into the cavernous sinus and prepontine trigeminal nerve. The lumbar puncture revealed a mononucleocytosis; however, the CSF PCR was negative for VZV, likely due to being already on antiviral therapy. CSF findings of an aseptic meningitis are more common in HZO patients with more severe manifestations such as complete ophthalmoplegia [5]. Subclinical extension of VZV into the CNS is also common, as CSF leukocytosis has been found in 46% of patients with VZV without clinical signs of meningitis or encephalitis [6].

Most patients show improvement over a few months in orbital signs and symptoms following treatment with antivirals. Over three in four patients completely recover from ophthalmoplegia and optic neuritis at a mean follow-up of 4.4 months; however, around 45% of patients may be left with residual ptosis [5, 7]. In addition, postherpetic neuralgia (PHN) remains one of the most common and disabling sequelae of HZO. Our patient did not report persistent neuropathic pain, but the risk of PHN is important to recognise and treat. Treatment with antivirals may also show resolution of the increased signal intensity within the brainstem or the cranial nerves that were affected [8, 9]. There is no consensus regarding the use of steroids in patients with VZV-related myositis, although the majority of reported cases of VZV-related orbital inflammation have been treated with steroids [2, 10]. There is insufficient evidence as to whether the use of steroids can help to hasten recovery [7]. In our case, we commenced the patient on steroids following a negative PCR result on the CSF after seeking advice from the neurology team.

In summary, HZO may present with acute orbital inflammation and viral meningitis in immunocompetent patients. It can affect various orbital structures including the extraocular muscles, optic nerve sheath and lacrimal gland and may extend intracranially. Treatment involves intravenous antivirals, with possible use of systemic steroids. An improvement in clinical signs and symptoms is expected with treatment; however, some patients may be left with residual deficits.

## Data Availability

The data that support the findings of this study are available from the corresponding author upon reasonable request.
